# Transit and non‐transit 3D EPID dosimetry versus detector arrays for patient specific QA

**DOI:** 10.1002/acm2.12610

**Published:** 2019-05-13

**Authors:** Igor Olaciregui‐Ruiz, Begoña Vivas‐Maiques, Jochem Kaas, Thijs Perik, Frits Wittkamper, Ben Mijnheer, Anton Mans

**Affiliations:** ^1^ Department of Radiation Oncology The Netherlands Cancer Institute – Antoni van Leeuwenhoek Amsterdam The Netherlands

**Keywords:** detector arrays, EPID dosimetry, IMRT, patient specific QA, VMAT

## Abstract

**Purpose:**

Despite their availability and simplicity of use, Electronic Portal Imaging Devices (EPIDs) have not yet replaced detector arrays for patient specific QA in 3D. The purpose of this study is to perform a large scale dosimetric evaluation of transit and non‐transit EPID dosimetry against absolute dose measurements in 3D.

**Methods:**

After evaluating basic dosimetric characteristics of the EPID and two detector arrays (Octavius 1500 and Octavius 1000^SRS^), 3D dose distributions for 68 VMAT arcs, and 10 IMRT plans were reconstructed within the same phantom geometry using transit EPID dosimetry, non‐transit EPID dosimetry, and the Octavius 4D system. The reconstructed 3D dose distributions were directly compared by *γ*‐analysis (2L2 = 2% local/2 mm and 3G2 = 3% global/2 mm, 50% isodose) and by the percentage difference in median dose to the high dose volume (%∆HDV_D__50_).

**Results:**

Regarding dose rate dependency, dose linearity, and field size dependence, the agreement between EPID dosimetry and the two detector arrays was found to be within 1.0%. In the 2L2 *γ*‐comparison with Octavius 4D dose distributions, the average *γ*‐pass rate value was 92.2 ± 5.2%(1SD) and 94.1 ± 4.3%(1SD) for transit and non‐transit EPID dosimetry, respectively. 3G2 *γ*‐pass rate values were higher than 95% in 150/156 cases. %∆HDV_D__50_ values were within 2% in 134/156 cases and within 3% in 155/156 cases. With regard to the clinical classification of alerts, 97.5% of the treatments were equally classified by EPID dosimetry and Octavius 4D.

**Conclusion:**

Transit and non‐transit EPID dosimetry are equivalent in dosimetric terms to conventional detector arrays for patient specific QA. Non‐transit 3D EPID dosimetry can be readily used for pre‐treatment patient specific QA of IMRT and VMAT, eliminating the need of phantom positioning.

## 1 INTRODUCTION

The major recent advances in modern external beam radiation therapy, particularly since the arrival of VMAT, demand dosimetric methods and tools to perform patient specific QA in multiple planes or in 3D.^1^, ^2^ The only types of dosimeters currently capable of measuring full‐3D dose distributions are polymer gel dosimeters and radiochromic detectors.^3^, ^4^, ^5^, ^6^ A more popular option is the use of pseudo 3D dosimeters consisting of a multidimensional detector array capable of reconstructing a portion or the whole 3D dose distribution within a phantom. Commercially available detector arrays, such as ArcCHECK (Sun Nuclear Corp., Melbourne, FL), Octavius 4D (PTW, Freiburg, Germany), and Delta4 (Scandidos AB, Upsala, Sweden), compare measured to planned 3D dose distributions. Several studies have already investigated the dosimetric characteristics and performance of these devices.^7^, ^8^, ^9^, ^10^, ^11^, ^12^, ^13^, ^14^, ^15^ Although initially developed for patient setup verification, Electronic Portal Imaging Devices (EPIDs) also have useful dosimetric characteristics as summarized in review articles.^16^, ^17^ Some EPID‐based approaches already allow for patient specific QA by reconstructing 3D dose distributions within the patient or phantom anatomy. In 3D non‐transit EPID dosimetry, the dose is determined within the patient or phantom based on *in air* or *fluence* EPID measurements, that is, without an attenuating medium between the source and the detector.^18^, ^19^, ^20^ In 3D transit EPID dosimetry, the dose is reconstructed within the patient or phantom based on EPID measurements acquired behind the patient or phantom.^21^, ^22^, ^23^, ^24^, ^25^ A clear advantage of EPIDs is their availability which makes them suitable for large scale implementations. When used for patient specific QA, non‐transit 3D EPID dosimetry is easier and faster than transit 3D EPID dosimetry because it eliminates the need to position a phantom. Furthermore, in cases where non‐transit EPID dosimetry allows for 3D dose reconstruction within the patient anatomy, the need for phantom re‐planning is also eliminated.

One of the main reasons why EPIDs have not yet replaced detector arrays for patient specific 3D dose verification is that commercial solutions are not widely available yet. Furthermore, there is a lack of studies validating EPID dose distributions against absolute dose measurements in 3D. This is typically due to a combination of the cumbersome work required and software challenges. As a result, these studies usually comprise only a limited number of cases.^19^, ^20^, ^26^ The comparison with ion chamber measurements is feasible only in a limited number of points and hence insufficient for the validation of complex IMRT and VMAT plans. Consequently, 3D EPID‐based dosimetry solutions generally end up being validated against the TPS.

The aim of this study is to assess the accuracy of transit and non‐transit 3D EPID dosimetry versus detector arrays. After evaluating basic dosimetric characteristics of the EPID and two detector arrays (Octavius 1500 and Octavius 1000^SRS^), 3D dose distributions for 68 VMAT arcs, and 10 IMRT plans were reconstructed using EPID dosimetry and the Octavius 4D system. The reconstructed 3D dose distributions were directly compared by *γ*‐analysis and by differences in the median dose to the high dose volume. The clinical impact of replacing the Octavius 4D system by EPID dosimetry for patient specific pre‐treatment QA was also evaluated.

## 2 MATERIALS AND METHODS

### 2.1 Equipment

Measurements were performed on a VersaHD linear accelerator (Elekta AB, Stockholm, Sweden) equipped with an Agility MLC (5 mm leaf width). Also available was the MV imaging acquisition software iViewGT™ (Elekta AB, Stockholm, Sweden) with an amorphous silicon EPID (PerkinElmer XRD 1642 AP) which is situated at 160 cm distance from the linac target. The EPID panel has a detection area of 41 × 41 cm^2^ (1024 × 1024 pixels). Treatment plans with all available energies (6 MV, 10 MV, 6 MV FFF, and 10 MV FFF) were generated with Pinnacle V9.10 (Philips Medical Systems, Eindhoven, The Netherlands).

### 2.2 Detector arrays

The detectors used in this study were the Octavius 1500 and Octavius 1000^SRS^ 2D arrays (PTW, Freiburg, Germany). The 1500 array has an active area of 27 × 27 cm^2^ with a center‐to‐center detector spacing of 7.1 mm. The 1000^SRS^ array is typically used for small target plans due to its smaller active area and higher resolution. In this array, the center‐to‐center detector spacing is 2.5 mm in an area of 5.5 cm × 5.5 cm around the center and 5 mm in the remaining outer area (up to 11 cm × 11 cm). In both cases the reconstructed dose cube has a 2 mm grid. A detailed characterization of both devices can be found elsewhere.^27^, ^28^ The arrays are inserted in the Octavius 4D phantom which is a cylindrical phantom made of water‐equivalent white polystyrene (RW3) with a slot to hold the detector array. An inclinometer is placed on the gantry, this is connected to a control unit that sends the movement information to the phantom. This allows the detector to rotate synchronously with the gantry ensuring that the detector remains perpendicular to the beam at all times during delivery. Based on these measurements, 3D dose distributions can be reconstructed within the Octavius 4D phantom geometry using the Verisoft 7.1 software (PTW, Freiburg, Germany). For both 2D detector arrays, a dose calibration prior to measurements is performed by delivering a 10 × 10 cm^2^ field per beam energy and providing the system with the expected dose.

### 2.3 3D transit EPID dosimetry

The commercially available iViewDose system (Elekta AB, Stockholm, Sweden) is a transit EPID dosimetry solution that uses EPID images acquired behind a patient in combination with CT data to reconstruct 3D dose distributions within the patient anatomy. Although intended for *in vivo* dose verification, iViewDose can also be used for pretreatment verification in combination with a phantom. Full details regarding the dose reconstruction can be found in the original design of the algorithm.^21^, ^29^ For IMRT verification, the iViewGT acquisition software (Elekta AB, Stockholm, Sweden) averages the total signal of all EPID frames between beam‐on and beam‐off into one accumulated portal image. The reconstructed 3D dose distributions of all beams are then summed to obtain the 3D dose distribution of the IMRT fraction. For VMAT verification, cine‐mode image acquisition is used and separate EPID frames are continuously being acquired during delivery. Each recorded frame is associated with a gantry angle. The reconstructed 3D dose distributions of all frames are then summed to obtain the 3D dose distribution of the total VMAT arc.

In order to determine the accuracy of transit EPID dosimetry, use was made of a unique experimental setup. Transit EPID images were acquired behind an Octavius 4D phantom with an homogeneous insert, that is an entirely RW3‐filled phantom. A CT scan of this Octavius phantom was available for both planning and dose reconstruction of the EPID transit images. Regarding the calibration of the iViewDose system, a full commissioning procedure was performed for each energy. The process involves the acquisition of *in air* EPID images and transit EPID images behind phantoms of varying thickness made of 30 × 30 cm^2^ sheets of RW3 for a variety of square fields (100 MU) delivered at maximum dose rate. The numerical values of the model parameters of the back‐projection algorithm are determined by fitting results to absolute dose measurements. The whole process takes about 3 h per energy. iViewDose uses a simple 0D couch attenuation model that also has to be comissioned for each energy.^30^

### 2.4 3D non‐transit EPID dosimetry

A research version of the same iViewDose package allows for non‐transit EPID dosimetry in 3D. The non‐transit algorithm uses *in air* EPID measurements in combination with CT data to determine a “virtual” transit primary portal dose distribution at the EPID level. Virtual transit primary portal dose distributions predict the transit primary portal dose distributions that would be measured behind a patient without changes in anatomy. In IMRT reconstructions, the non‐transit algorithm uses accumulated *in air* portal images to estimate the accumulated “virtual” transit primary portal dose distribution corresponding to each field. In VMAT reconstructions, the non‐transit algorithm uses *in air* portal arcs to estimate the “virtual” transit primary portal dose distribution of each VMAT frame. These virtual transit primary portal dose distributions are used in combination with patient CT data to reconstruct 3D dose distributions directly within the patient anatomy. This method is therefore very useful for patient specific pretreatment QA since it provides information about the dose to be delivered to the patient, including potential machine and planning errors. Note that apart from the determination of the primary portal dose distribution, the transit and non‐transit dose reconstruction algorithms are identical. In order to determine the accuracy of non‐transit EPID dosimetry in this study, *in air* EPID measurements were used in combination with the CT data set of the adapted Octavius 4D phantom to reconstruct non‐transit 3D dose distributions within this phantom geometry. Full details regarding the non‐transit dose reconstruction engine can be found elsewhere.^19^

### 2.5 Dosimetric characterization

**Figure 1:**
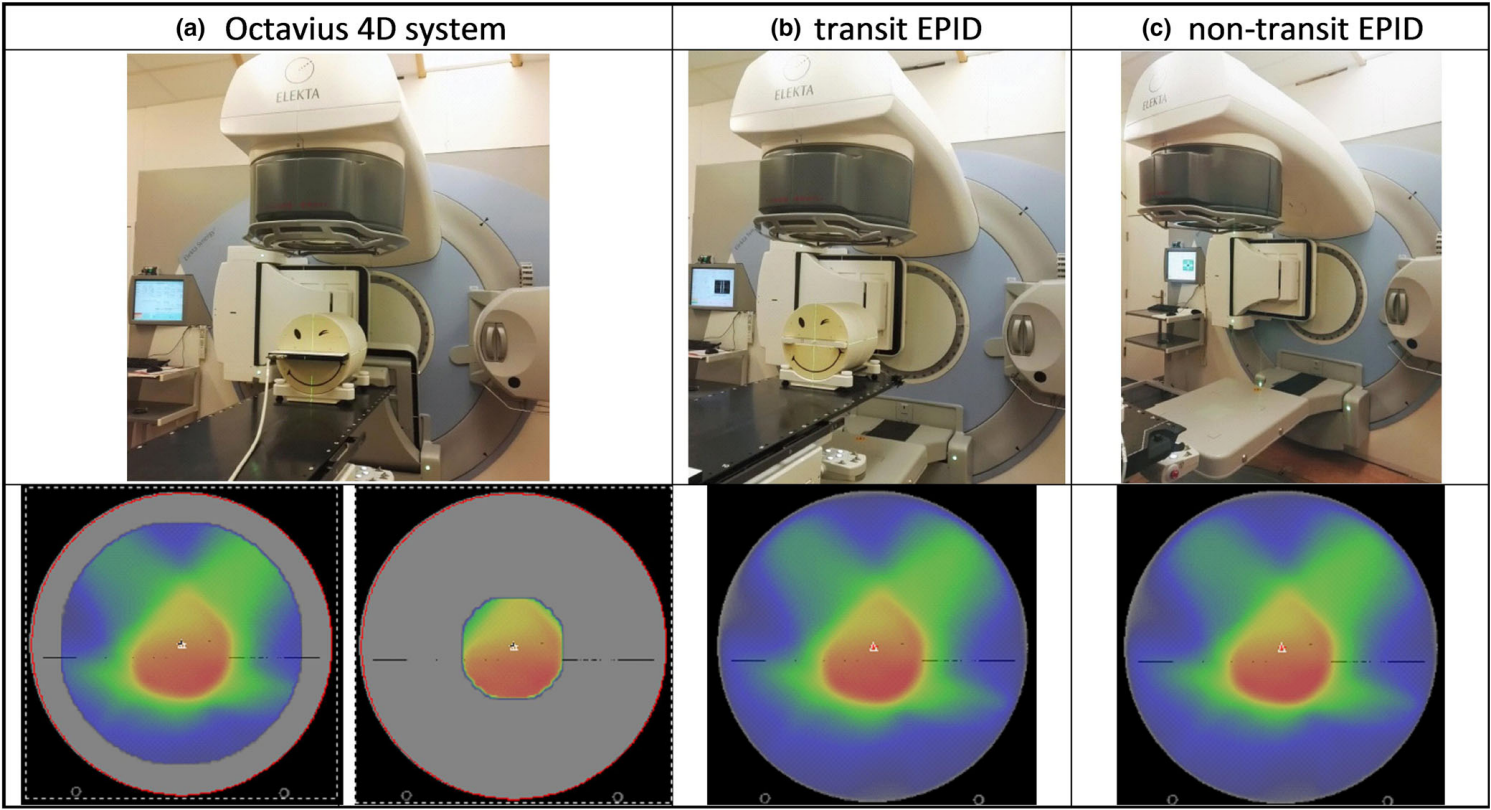
Schematic diagram of the measurement configurations used in this study to reconstruct 3D dose distributions within the Octavius 4D phantom geometry: (a) the Octavius 4D phantom with the Octavius 1500 or with the Octavius 1000^SRS^ 2D detector array, (b) the transit EPID dosimetry setup with the adapted Octavius 4D phantom, and (c) the non‐transit EPID dosimetry set‐up.

The dose rate dependency was evaluated, for each combination of measurement configuration and energy shown in Fig. 1, by irradiating a total of 100 MU for a 10 × 10 cm^2^ field at five different dose rates (100%, 50%, 25%, 12.5%, and 6% of the maximum dose rate). For each energy, the ratio of the reading to MU delivered was calculated and normalizsed to the maximum dose rate. The dose linearity was studied over the range 5–1000 MU for flattened beams and 10–1000 MU for unflattened beams. The ratio of the reading to MU delivered was calculated and normalised to 100 MU in each case. All measurements were made at gantry 0°. Note that the Octavius 1000^SRS^ presents a non‐negligible dependence on dose rate as well as on field size.^31^, ^32^ The manufacturer of the Octavius 1000^SRS^ array recommends to cross‐calibrate the detector at a 4 × 4 cm^2^ field size to mitigate the overall effect of the field size dependence. In our clinic, the detector is calibrated at a 10 × 10 cm^2^ field size and an in‐house implemented correction is applied to each measurement frame prior to the reconstruction. In this correction, the actual effective field size and dose rate are first determined from the data of each measurement frame, and then a correction factor for that field size and also for the dose rate is applied on a per‐frame basis. The correction factors are empirically determined by comparing measurements performed with the detector array to reference data.

### 2.6 Square fields

For each measurement configuration and energy, 3D dose distributions were reconstructed for a set of square fields irradiated at gantry 0° at maximum dose rate. The width of the fields ranged from 3 to 20 cm for flattened beams and from 3 to 10 cm for unflattened beams. The isocenter dose values were compared with extra measurements performed within the Octavius 4D phantom with a microDiamond solid detector detector (PTW Freiburg, Germany) which were used as reference. 2D dose distributions at the isocenter plane were compared by using *γ*‐analysis and by visual inspection of cross‐plane dose profiles. *γ*‐analysis was performed using a strict set of *γ*‐criteria of 2% local/2 mm (2L2) with statistics calculated within the area surrounded by the isodose line defined by 20% of the maximum planned dose.

### 2.7 IMRT and VMAT treatments

For each measurement configuration, 3D dose distributions were reconstructed for 10 IMRT plans and 68 VMAT arcs (34 plans). The Octavius 1000^SRS^ array was used for the smaller target volumes and the Octavius 1500 array for the larger ones. The Octavius 1000^SRS^ 3D dose reconstructions were corrected for dose rate and field size, as routinely applied in our clinic. The plans were randomly selected from our clinical database. These included prostate, rectum, stereotactic lung, stereotactic brain and head‐and‐neck VMAT treatments, and lung IMRT treatments. The Verisoft software saved the Octavius 1500 and Octavius 1000^SRS^ dose volumes as dicom (RTDOSE) files. These were imported in the research version of iViewDose for a direct comparison with EPID dose distributions. The comparison was performed by using *γ*‐analysis with two sets of *γ*‐criteria: the strict 2L2 set (2% local/2 mm) and a more lenient 3G2 set (3% global/2 mm). Statistics were calculated within the volume surrounded by the isodose surface defined by 50% of the maximum planned dose. This rather high threshold value was chosen to avoid artificial improvement of the global *γ* evaluation results by including low dose volumes. Note also that this is the current threshold of choice at our institute for patient specific dosimetric verification in 3D, both for pretreatment and *in vivo*. To gain insight on the differences in absolute dose values, the median dose to the high dose volume (HDV_D50_) was also used as comparison metric between the distributions. The HDV is the volume surrounded by the isodose surface defined as 80% of the maximum planned dose.

### 2.8 Comparison with TPS: alert classification rate

Patient specific QA in 3D is usually carried out by comparing the dose distribution measured in a phantom to the planned dose distribution recalculated on the phantom geometry. The comparison between the measured and the planned dose distributions is typically performed by *γ*‐analysis. A recent AAPM TG‐218 report recommended the use of 3G2 *γ*‐pass rate as alert indicator for patient specific IMRT QA, and proposed tolerance and action levels of 95% and 90%, respectively.^33^ A treatment is classified either as positive (alerted) or negative (not alerted) depending on whether the 3G2 *γ*‐pass rate value falls below or above the threshold level, respectively. To compare the clinical performance of each measurement configuration in this study for patient specific QA, the reconstructed 3D dose distributions (with transit EPID dosimetry, non‐transit EPID dosimetry, and Octavius 4D system) were compared with the dose distributions calculated with our planning system in the Octavius 4D phantom. Pinnacle V9.10 uses a Collapsed Cone Convolution Superposition (CCCS) algorithm to determine dose distributions. The TPS dose calculations in this study were performed using a dose grid resolution of 2 mm. The rate of equal alert classification between the various measurement configurations was evaluated.

## 3 RESULTS

### 3.1 Dosimetric characterization

**Figure 2:**
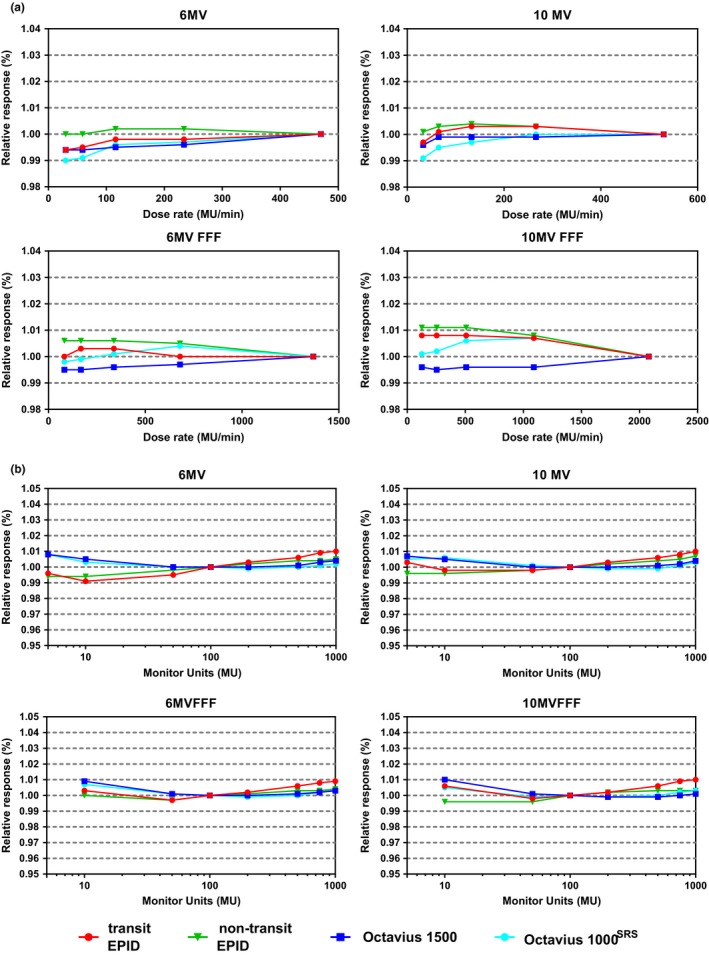
(a) Dose rate dependence and (b) dose linearity for the measurement configurations used in this study. Results are presented separately for each beam energy and normalized to the maximum dose rate in (a) and to 100 MU in (b).

All detectors studied in this work presented a dependency on dose rate, see Fig. 2a. This dependency varied per energy. Deviations were within 1.0% for all detectors. As can be seen in Fig. 2b, the dose linearity was within 1.0% between 5 and 1000 MU for flattened beams and between 10 and 1000 MU for unflattened beams for all detectors for all energies. No saturation effects were observed with non‐transit EPID dosimetry.

### 3.2 Square fields

**Figure 3:**
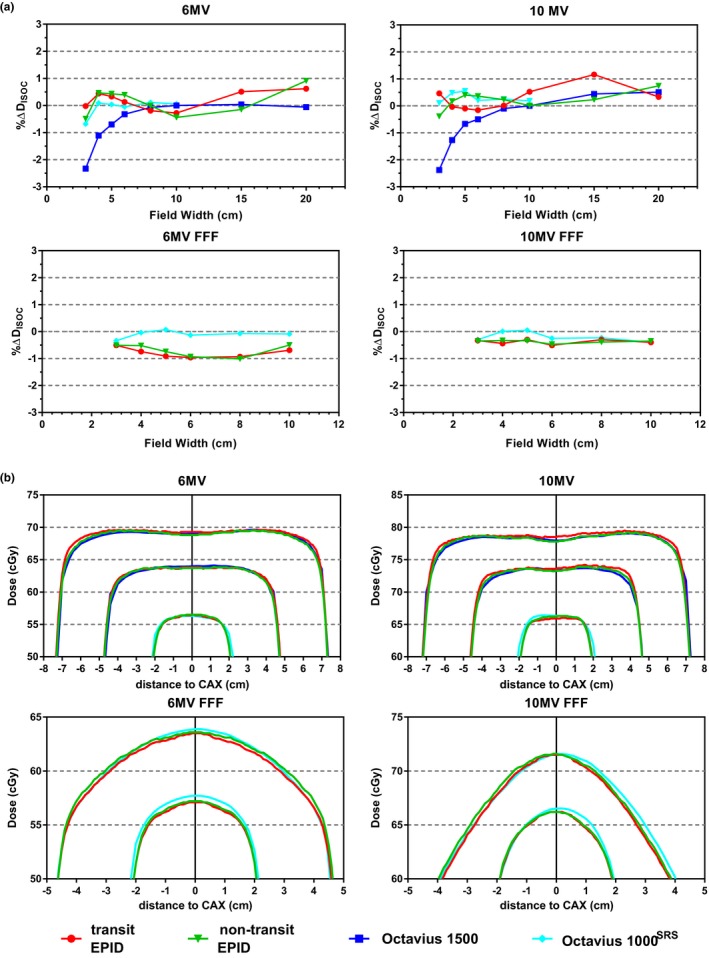
(a) Difference in isocenter dose values between each measurement configuration and reference measurements performed with a microDiamond solid state detector for a set of field sizes. (b) Octavius 1500, Octavius 1000^SRS^, transit EPID dosimetry, and non‐transit EPID dosimetry cross‐plane profiles through the isocenter plane of reconstructed 3D dose distributions for a set of field sizes. Results are presented separately for each beam energy.

Fig. 3a displays the difference in isocenter dose values between the measurement configurations and the reference measurements performed with the microDiamond detector. Transit and non‐transit EPID‐reconstructed isocenter dose values were within 1% of the reference value. The same applied to the Octavius arrays, except for the Octavius 1500 array at field widths smaller than 4 cm. This volume effect of the Octavius 1500 array has already been reported in the literature.^34^ The average difference with Octavius isocenter dose values was −0.1 ± 0.5(1 SD) and −0.1 ± 0.4(1 SD) for transit and non‐transit EPID dosimetry, respectively. The agreement between cross plane profiles through the isocenter was excellent, as exhibited in Fig. 3b. In the 2L2 *γ*‐comparison with Octavius 2D dose distributions at the isocenter plane, the average *γ*‐pass rate value was 96.5 ± 1.8%(1 SD) and 97.6 ± 2.3%(1 SD) for transit and non‐transit EPID dosimetry, respectively. The average *γ*‐mean value was 0.41 ± 0.06(1 SD) and 0.39 ± 0.09(1 SD), respectively.

### 3.3 IMRT and VMAT treatments

**Figure 4:**
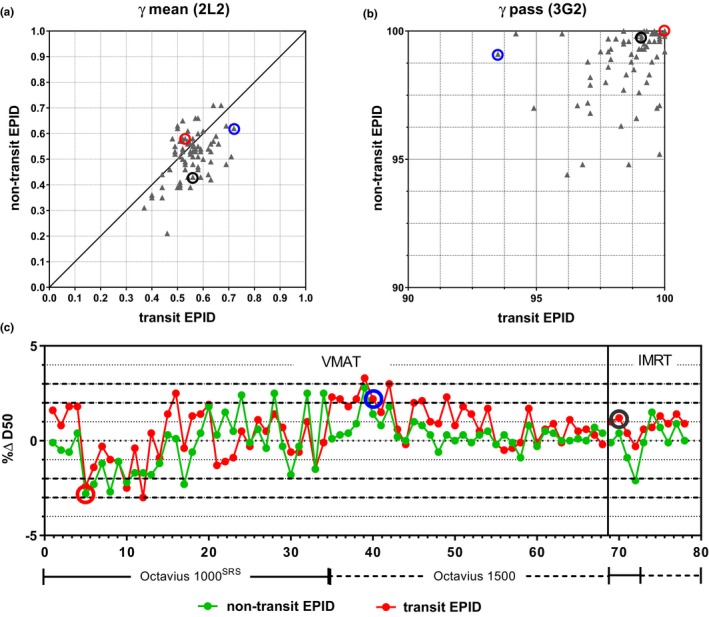
Scatter plots displaying results of the direct comparison between 3D dose distributions reconstructed with Octavius 4D and the corresponding 3D dose distributions reconstructed with transit and non‐transit EPID dosimetry using (a) 2L2 *γ*‐mean values, (b) 3G2 *γ*‐pass rate values, and (c) percentage difference in median dose to the high dose volume %ΔHDV_D__50_ (Octavius 4D used as reference). The VMAT cases in (c) are arranged in increasing order of analyzed volume. The circled data points correspond to a 6MV head‐and‐neck VMAT plan that showed the largest 2L2 *γ*‐mean value (blue), to a 6MV FFF VMAT stereotactic brain plan that presented one of the largest deviations in HDV_D__50_ (red) and to a 10MV IMRT lung case (black).

The results of the direct comparison between the Octavius 4D system and EPID dosimetry are presented in Table 1. Results are presented as Average ± (1 SD) with the range indicated between parenthesis. Two sets of *γ*‐criteria are used in the comparison, a strict 2L2 set (2% local/2 mm) and a more lenient 3G2 set (3% global/2 mm). Note that the percentage difference in median dose to the high dose volume (%∆HDV_D50_) uses the Octavius 4D value as reference. In the 2L2 *γ*‐comparison with Octavius 3D dose distributions, the average *γ*‐pass rate value for VMAT and IMRT combined was 92.2 ± 5.2%(1 SD) and 94.1 ± 4.3%(1 SD) for transit and non‐transit EPID dosimetry, respectively. The average 2L2 *γ*‐mean value was 0.55 ± 0.07%(1 SD) and 0.51 ± 0.09%(1 SD), respectively. A scatter plot exhibiting all 2L2 *γ*‐mean values is shown in Fig. 4a. An equivalent scatter plot displaying all 3G2 *γ*‐pass rate values is also displayed in Fig. 4b. The average 3G2 *γ*‐pass rate value was 98.7 ± 1.4%(1 SD) and 98.9 ± 1.4%(1 SD) for transit and non‐transit EPID dosimetry, respectively. 3G2 *γ*‐pass rate values were higher than 95% in 150/156 cases. Fig. 4c displays the percentage differences in HDV_D50_ values with the VMAT cases displayed in increasing order of analyzed volume size. Differences were within 3% in 155/156 cases and within 2% in 134/156 cases.

**Table 1:** Direct comparison of 3D dose distributions reconstructed by the Octavius 4D system and EPID dosimetry

Octavius 4D vs		Transit EPID dosimetry			Non‐transit EPID dosimetry		
*γ*‐criteria	# cases	*γ*‐mean	*γ*‐pass %	%∆HDV_D50_	*γ*‐mean	*γ*‐pass %	%∆HDV_D50_
2L2	68 VMAT	0.55 ± 0.08 (0.37, 0.72)	92.3 ± 5.5 (78.5, 99.4)	0.6 ± 1.3 (−3.0, 3.3)	0.51 ± 0.10 (0.21, 0.71)	94.2 ± 4.4 (79.4, 99.5)	0.0 ± 1.2 (−2.8, 2.8)
	10 IMRT	0.55 ± 0.03 (0.49, 0.59)	91.7 ± 2.3 (87.4, 95.9)	−0.8 ± 0.5 (−1.4, 0.3)	0.52 ± 0.05 (0.43, 0.56)	93.1 ± 2.9 (89.4, 98.4)	−0.1 ± 0.9 (−1.5, 1.6)
3G2	68 VMAT	0.37 ± 0.07 (0.21, 0.51)	98.7 ± 1.4 (93.5, 100)		0.34 ± 0.09 (0.17, 0.51)	99.0 ± 1.4 (94.4, 100)	
	10 IMRT	0.38 ± 0.02 (0.35, 0.42)	98.5 ± 0.9 (96.6, 99.7)		0.36 ± 0.05 (0.28, 0.43)	98.3 ± 1.2 (96.6, 99.8)	

**Figure 5:**
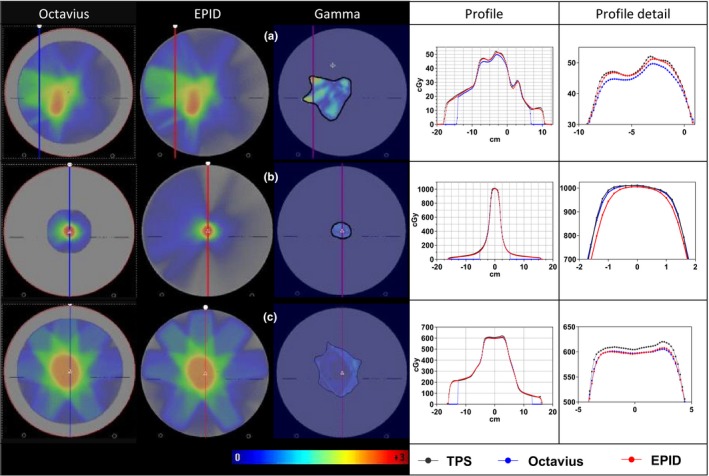
3D dose reconstructions, 2L2 *γ*‐distributions, and dose profiles in the direct comparison between (a) transit EPID dosimetry and Octavius 4D 1500 for the 6MV head‐and‐neck VMAT plan that showed the largest 2L2 *γ*‐mean value, (b) non‐transit EPID dosimetry and Octavius 4D 1000^SRS^ for a 6MV FFF VMAT hypo brain plan that presented one of the largest deviations in HDV_D__50_, and (c) transit EPID dosimetry and Octavius 4D 1500 for a 10MV IMRT lung plan. The 2L2 *γ*‐mean values in (a), (b), and (c) were 0.72, 0.58, and 0.56, respectively. The differences in HDV_D__50_ values between EPID dosimetry and Octavius 4D were 2.2%, −2.8%, and 1.2%, respectively.

The highest 2L2 *γ*‐mean value (0.72) was found for a VMAT head‐and‐neck case, indicated by the blue circled data points in Fig. 4. This case presented also the lowest 3G2 *γ*‐pass rate (93.5%). Graphical data corresponding to this analysis are exhibited in Fig. 5a. As can be seen in the profiles, EPID dosimetry showed a better agreement with the TPS than Octavius 4D. The red circled data points in Fig. 4 correspond to a 6MV FFF VMAT stereotactic brain plan which presented one of the largest HDV_D50_ differences (−2.8%). The underdosage with EPID dosimetry is further displayed in the zoomed parts of Fig. 5b. However, the 2L2 *γ*‐mean value for this comparison was 0.56 which illustrates the insufficiency of *γ*‐evaluation alone to compare dose distributions in high dose gradient volumes. In this case, Octavius 4D presented a better agreement with the TPS than EPID dosimetry. For completeness, an IMRT lung case is displayed in Fig. 5c where EPID dosimetry and Octavius showed a better agreement among themselves than with the TPS.

### 3.4 Comparison with TPS: alert classification rate

**Figure 6:**
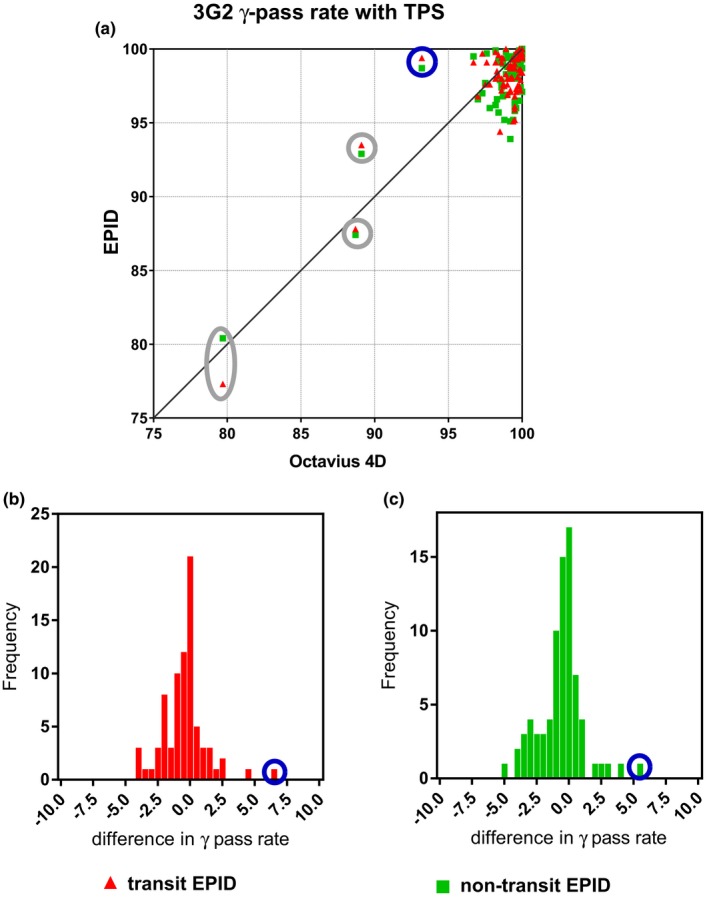
(a) Scatter plots comparing the agreement between dose distributions reconstructed with EPID dosimetry and the Octavius 4D system and the corresponding planned dose distributions in terms of 3G2 *γ*‐pass rate values and (b,c) histograms of differences in TPS comparison results between EPID dosimetry and Octavius also expressed in terms of 3G2 y pass rate values.

Figure 6a exhibits a scatter plot with 3G2 *γ*‐pass rate values corresponding to the comparison between the reconstructed 3D dose distributions of this study (with transit EPID dosimetry, non‐transit EPID dosimetry, and Octavius 4D system) and the corresponding dose distributions calculated by the planning system. In the 3G2 *γ*‐comparison with planned 3D dose distributions, the average *γ*‐pass rate value was 98.6 ± 2.9%(1 SD), 98.0 ± 3.0%(1 SD), and 97.9 ± 2.8%(1 SD) for Octavius 4D, transit EPID dosimetry, and non‐transit EPID dosimetry, respectively. Using the recommended tolerance level of 95% as *γ*‐pass rate alert threshold value, 76 out 78 cases were equally flagged by EPID dosimetry (both transit and non‐transit) and Octavius 4D. Figure 6b and c show histograms of the differences in 3G2 *γ*‐pass rate values between EPID dosimetry (transit and non‐transit) and Octavius 4D in their comparison with dose distributions calculated by the planning system. The average difference was −0.6 ± 1.7%(1 SD) and −0.8 ± 1.7%(1 SD) for transit and non‐transit EPID dosimetry, respectively. The largest differences, circled in blue in Fig. 6, corresponded to the 6 MV head‐and‐neck VMAT case of Fig. 5a. This case was flagged as a positive by the Octavius 4D system with a 3G2 *γ*‐pass rate of 93.2%. Transit and non‐transit EPID dosimetry presented a considerably better agreement with the TPS, 99.4% and 98.7%, respectively. The Octavius measurements were repeated six times with different dose rates yielding similar results. This head‐and‐neck is a singular case in which a highly modulated VMAT partial arc (40°–178°) delivers most of the MU's in a few small ranges of gantry angles. Since fewer gantry angles are sampled we expect that the spatial resolution of the 1500 detector will cause problems in this particular case. The other VMAT head‐and‐neck measurements included in this study corresponded not only to less modulated cases but were also delivered as full arcs where the effects of the lack of spatial resolution are expected to smooth out. There were three more cases, circled in gray in Fig. 6a, where a poor agreement with the plan was obtained with both Octavius 4D and EPID dosimetry. These cases are further discussed in the discussion section.

## 4 DISCUSSION

In this study, we have demonstrated that transit and non‐transit 3D EPID dosimetry show similar dosimetric characteristics and accuracy to detector arrays. The results presented in Section 3.A show that dose rate dependency and dose linearity differences are within 1% for clinically relevant situations. This is important since it can be concluded that the EPID dosimetry algorithm does not require corrections for these effects. The results of this study indicate an excellent dosimetric agreement between the 3D dose distributions reconstructed with EPID dosimetry and Octavius 4D. Average 2L2 *γ*‐mean values below 0.6 suggest that, on average, a dose difference of ~1.2% is expected in low‐dose gradient regions or a DTA of ~2 mm in steep dose gradient regions. The average 3G2 *γ*‐pass rate value found for the 10 IMRT plans and 68 VMAT arcs was 98.8%. For comparison, a recent study comparing 3D dose distributions reconstructed with Octavius 4D and with non‐transit EPID dosimetry reported 3G3 *γ*‐pass rates of 95% and 93% for one IMRT prostate plan and one VMAT head‐and‐neck plan, respectively.^20^ Also, in the initial investigation of the non‐transit dose reconstruction algorithm used in this study,^19^ an average 3G3 *γ*‐pass rate value of 98.5% was reported for 5 IMRT and 5 VMAT plans. In another recent study, the EPID‐reconstructed 3D dose distribution for one VMAT lumbar spine vertebra was compared with gel dosimetry reporting a 3G3 *γ*‐pass rate value of 93%.^26^

As can be seen from the 2L2 *γ*‐results in Table 1, non‐transit EPID dosimetry slightly outperforms transit EPID dosimetry in the comparison with Octavius. This might be expected from the results of the validation of the commissioning models in Section 3.B where non‐transit EPID dosimetry presented higher 2L2 *γ*‐pass rate values than transit EPID dosimetry. Note that the transit and non‐transit EPID dosimetry reconstruction algorithms are commissioned separately for each energy and hence, small differences in the parameters of these models may result in small differences in the reconstructed doses. An additional explanation could be that the determination of the transit primary portal dose distribution at the EPID level requires corrections for the couch attenuation and the patient scatter which are not needed in the determination of “virtual” transit primary portal dose distributions. Figure 5b also highlights the particular challenge of EPID dosimetry for techniques that combine the use of unflattened beams and small field sizes such as stereotactic VMAT brain. We are currently in the process of modifiying our commissioning process to improve the accuracy of EPID dosimetry for small fields.

Since patient specific IMRT and VMAT QA is performed by comparing measured and planned dose distributions, the performance of measuring devices such as detector arrays or EPIDs is typically evaluated in the literature by their comparison to the TPS. The 3D EPID dosimetry and Octavius 4D results presented in Section 3.D and in Fig. 6a agree well with previous publications on patient specific IMRT and VMAT QA.^8^, ^11^, ^13^, ^14^, ^35^, ^36^, ^37^ Figure 6a helps evaluating the clinical impact of replacing detector arrays by EPID dosimetry for patient specific QA. With the recommended 3G2 tolerance level of 95% as threshold level for alert classification, the percentage of equal treatment classification between EPID dosimetry and Octavius 4D was 97.5%. At our institute, pretreatment patient specific QA is already performed by non‐transit 3D EPID dosimetry, and only when deviations are detected that cannot be explained, we make use of Octavius 4D as an alternative verification device. A shortcoming of EPIDs is their inadequacy to acquire large clinical fields that may irradiate the EPID electronics. In these cases, the verification is performed with a derivative plan with reduced field size. Damage to the panel is an issue that has to be considered when using EPIDs on a regular basis since it has an influence on the lifetime of the detector. A weekly QA procedure monitors the long‐time reproducibility of the panel response with an output measurement. This process is fully automated in our clinic.

Particularly relevant are the situations where EPID dosimetry and Octavius show a better agreement among themselves than with the TPS. An explanation could be that the choices made during TPS commissioning were suboptimal for these particular type of plans. Another explanation could be the effect that the gantry spacing resolution has on the accuracy of the TPS dose calculations. In practice, actions in the clinic are taken depending on the magnitude of the detected deviation. In cases where the physician judges the deviation to be too large to be clinically acceptable, a practical solution could be the creation of new (less complex or interpolated) plans. For instance, the data points circled in gray in Fig. 6a corresponded to plans with a high degree of complexity, where the gantry spacing resolution has a non‐negligible effect on the accuracy of the dose calculations. In two of the three cases, the presented data corresponded to calculations using a gantry spacing of 4°. In the third one, a gantry spacing of 2° was used. For the plans with 4° gantry spacing, new plans with 2° gantry spacing were calculated. After this increase in gantry spacing resolution, the 3G2 *γ*‐pass rate values become higher than 95% except for one case with Octavius 1500, possibly due to the same spatial resolution issues as in the case circled in blue in Fig. 6. In the plan calculated with 2° gantry spacing, most of the dose is delivered in two peaks within a 20° degree gantry angle value range. Profiles of the TPS versus any of the detectors presented low peaks across the high dose region and high valleys across the low dose region. Unfortunately, an increase in the gantry spacing resolution to 1° is not allowed by the planning system.

The transit 3D EPID dosimetry system is clinically used for *in vivo* dose verification of all our external photon beam treatments. The high accuracy shown by transit 3D EPID dosimetry in this study confirms the reliability of the method. *In vivo* dose verification presents, however, extra challenges and hence, additional uncertainties. First, the parameters of the dose reconstruction algorithm are determined using water‐based scatter correction kernels and consequently, the model does not account for tissue inhomogeneities accurately. Therefore, for dose verifications of sites involving (large) tissue heterogeneities, the *in aqua vivo* approach must be used.^38^ Second, the dose delivered to the patient during treatment is affected by patient setup variations and patient anatomical changes.^39^ Last, another limitation of the algorithm is that it uses the planning CT as patient anatomy model for dose reconstruction. To better cope with the challenge of patient variations, more accurate results are expected once the daily patient anatomy is used in the reconstruction.

## 5 CONCLUSION

Transit and non‐transit EPID dosimetry are equivalent in dosimetric terms to conventional detector arrays for patient specific QA. Non‐transit 3D EPID dosimetry can be readily used for pretreatment patient specific QA of IMRT and VMAT, eliminating the need of phantom positioning.

## CONFLICT OF INTEREST

Our department licenses software for portal dosimetry to Elekta Oncology Systems Ltd. The authors are responsible for the content and writing of the paper.
